# Patient-reported outcomes after free muscle flap coverage for therapy-resistant neuropathic pain from the ulnar nerve

**DOI:** 10.1177/17531934231201930

**Published:** 2023-09-25

**Authors:** Emile B. List, Nadine Boers, Enrico Martin, David D. Krijgh, J. Henk Coert

**Affiliations:** Department of Plastic and Reconstructive Surgery, University Medical Center Utrecht, Utrecht, The Netherlands

**Keywords:** Free flap, neuropathic pain, upper extremity

## Abstract

Extensive microsurgical neurolysis followed by free gracilis muscle flap coverage can be performed as a last resort for patients with persistent neuropathic pain of the ulnar nerve. All patients who had this surgery between 2015 and 2021 were identified. Data were collected from the medical records of 21 patients and patient-reported outcomes were collected from 18 patients, with a minimum follow-up of 12 months. The median visual analogue pain score decreased significantly 8 months postoperatively from 8.0 to 6.0 and stabilized to 5.4 at the 3-year follow-up. Health-related quality-of-life scores remained diminished compared to normative data. In the treatment of therapy-resistant neuropathic pain of the ulnar nerve, extensive neurolysis with a subsequent free gracilis muscle flap coverage shows a promising reduction of pain that persists at long-term follow-up.

**Level of evidence:** IV

## Introduction

Surgical decompression of the ulnar nerve is successful in most patients experiencing ulnar compression neuropathy (Wade et al., 2020). In 10%–25% of patients, however, symptoms persist or worsen, and may lead to a chronic neuropathic pain syndrome (Wade et al., 2020). Ulnar nerve submuscular transposition may improve symptoms after failed primary decompression, but 15% of patients do not respond (Boers et al., 2022). Neuropathic pain is known to have a significant negative impact on physical functioning and quality of life (Gilron et al., 2006; Stokvis et al., 2010a), and treatment often involves multiple modalities, such as opioids, anti-depressants, anxiolytics, neuro-stimulation devices, nerve blocks and multiple revision surgeries (Dahlin et al., 2022; Vij et al., 2020). Currently, there is no optimal treatment option for patients experiencing persistent ulnar nerve neuropathic pain, resulting in some patients experiencing residual pain despite numerous operations and medical treatment.

Extensive neurolysis with free muscle flap coverage, as previously described by the senior author of this paper, can be a last resort in this situation (van Bekkum et al., 2020). The objective is to create a scar-free, traction-free, well-padded and well-vascularized environment for the nerve. The previous study was a small case series that demonstrated long-term pain reduction postoperatively. The limitations of this case series were that the patients included were heterogeneous (patients with radial, median, ulnar and digital neuropathies were included) and that there was a relatively high flap loss reported within this series. This aim of the present study was to provide additional insights on postoperative outcomes by utilizing a new dataset of patients with long-term follow-up.

## Methods

### Study design

This was a retrospective and cross-sectional study, using data collected from all patients who underwent an extensive microsurgical neurolysis followed by free gracilis flap coverage for the treatment of persistent neuropathic pain of the ulnar nerve between January 2015 and November 2021. All surgeries were performed by the senior author of this paper (JHC) using a previously described surgical technique (van Bekkum et al., 2020). Ethical approval for this study was obtained from the Medical Research Ethics Committee (22-918).

### Patient selection and recruitment

Patients were eligible for inclusion if they met the following criteria: (1) revision neurolysis with free gracilis flap coverage for persistent neuropathic ulnar nerve pain; (2) aged 18 years or older at the moment of free flap reconstruction; and (3) follow-up of at least 12 months after the reconstruction. Exclusion criteria were a mental or physical inability to read, understand and/or complete the questionnaires. All patients were recruited in November 2022. Eligible patients were informed about the study by phone. Written informed consent was obtained from all participants before the study. Patients completed the questionnaires using Castor EDC, a secured web-based clinical data management platform.

### Demographics

Patient-related characteristics were recorded from the electronic medical records. These included sex, age, body mass index, co-morbidities, medical history and visual analogue scale (VAS) score for pain. Surgery-related characteristics included time since reconstruction, time between the previous surgery and the reconstruction, and the affected side. The surgical outcomes that were retrieved included length of stay, complications and VAS scores.

### Patient-reported outcome measures (PROMs)

PROMs were measured using four questionnaires, a pain score, and two questions on pre- and postoperative pain medication. All PROMs were validated and translated into Dutch. Pain in general was evaluated using a VAS score, with scores in the range of 0–100. General health was assessed using the 36-Item Short-Form Health Survey (SF-36), a widely used health-related quality-of-life questionnaire consisting of 36 items divided into eight scales (Ware and Sherbourne, 1992). The scores range from 0 to 100, with higher scores representing better physical and mental well-being. The summary scores Physical Component Score (PCS) and Mental Component Score (MCS) were calculated from the eight domains using normative data from a Dutch population combined with United States factor score coefficients (Aaronson et al., 1998; Ware et al., 1993). Quality of life was assessed using the five-level EuroQol-5 Dimension (EQ-5D-5L) instrument (Herdman et al., 2011). This questionnaire comprises five dimensions: mobility; self-care; usual activities; pain/discomfort; and anxiety/depression. For the SF-36 and EQ-5D-5L scores, subgroups were created based on unilateral neuropathic pain or bilateral neuropathic pain of which only one side was surgically treated. Hand and arm function were assessed using the quick version of the Disabilities of the Arm, Shoulder, and Head questionnaire (QuickDASH) and the Michigan Hand Outcomes Questionnaire (MHOQ) (Beaton et al., 2005; Chung et al., 1998; Veehof et al., 2002). The Quick-DASH consists of 11 5-point Likert scale items. The responses were converted to an overall score in the range of 0–100, with higher scores indicating more disability. The MHOQ includes 57 5-point Likert scale questions on the following domains: overall hand function; daily activities; work activities; pain; aesthetics; and patient satisfaction regarding hand function. The scores range from 0 to 100, with higher scores representing better results. Scores of the reconstruction side were compared with the opposite extremity.

### Statistical analyses

Demographic and treatment variables were summarized using descriptive statistics and presented as medians with interquartile range or numbers. No imputations were used for missing data. Preoperative, postoperative in clinic and late postoperative median VAS scores were compared using a dependent sample non-parametric sign test. Patients with one or two prior surgeries to the reconstruction were compared to three or more previous operations using an independent sample non-parametric median test. The SF-36, EQ-5D-5L and MHOQ subgroup scores were compared using an independent sample Student’s *t*-test. Two-sided *p*-values <0.05 were considered statistically significant.

## Results

### Patient characteristics

Between January 2015 and December 2021, 24 reconstructions in 23 consecutive patients were identified. Two patients decided not to participate, leaving a total of 21 patients included in the study and 18 who responded to the questionnaires. One patient died from causes unrelated to the reconstruction, but data from their medical records were included in the analysis. One patient underwent bilateral reconstruction. All patients had previously undergone one or more operations on the affected upper extremity before the free muscle reconstruction, with a median of 3 operations and 2.1 years between last surgery and the free flap reconstruction. Out of 22 reconstructions, preoperative Tinel’s sign was positive in nine patients, negative in three and unknown in 10. All patient characteristics are shown in Table 1.

**Table 1.** Patient demographics.

	Study population
Patients	21
Sex	
Male	7
Female	14
Reconstructions	
Primary	22
Age (years)	50 (40–57)
BMI (kg/m^2^)	29 (24–32)
Co-morbidities	
Current smoker	4
Smoking history	6
Hypertension	6
Cardiovascular disease	0
Diabetes mellitus	0
Operations before free flap reconstruction	
1	2
2	9
3	4
4	3
5 or more	3
Unknown	1
Type of last surgery before free flap reconstruction	
Open decompression	4
Submuscular transposition	16
Denervation	1
Intraosseous transposition	1
Reconstruction on dominant side	
Yes	12
No	8
Unknown	2

Data are presented as n or median (IQR).

BMI: body mass index; IQR: interquartile range.

### Aesthetic results

**Figure 1.**
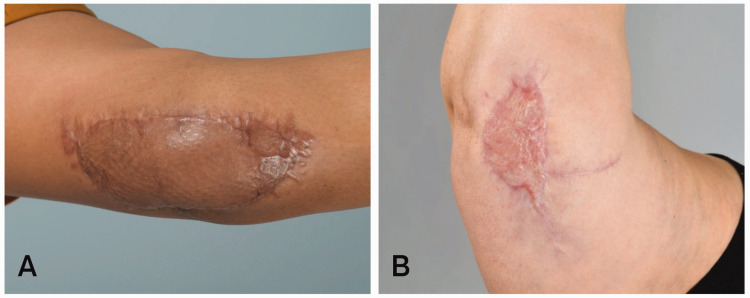
Postoperative aesthetic results. Postoperative aesthetic results of two patients treated for ulnar neuropathy. (a) The patient developed a wound infection in the second postoperative week. Photographs were taken 4 months after the procedure and (b) The patient had an uncomplicated recovery. Photographs were taken 3 months after the procedure.

The postoperative results of two patients are shown in Figure 1. The patient in Figure 1(a) developed a wound infection in the second postoperative week. The photograph was taken 4 months after the procedure and demonstrates that, despite the infection, the patient healed well. In Figure 1(b), the photograph was taken after an uncomplicated 3-month recovery.

### Complications

No flap loss or flap necrosis was observed in our patient cohort. All complications are shown in Table 2. One patient with operative neuropathic pain from the saphenous nerve was treated successfully with denervation. One patient who developed an infected seroma required incision and drainage.

**Table 2.** Surgery complications and hospital admission days.

	Reconstructions (n = 22)
Flap complication	
Flap necrosis	0
Vascular insufficiency	1
Postoperative haemorrhage	1
Wound dehiscence	1
Wound infection	0
Failure of skin graft take	0
Seroma	1
Donor site complication	
Wound infection	3
Abscess in scar tissue	1
Neuropathic pain saphenous nerve	1
Infected seroma	1
Admission days	5 (5–6)

Data are presented as n or median (IQR).

IQR: interquartile range.

### Pain

**Figure 2.**
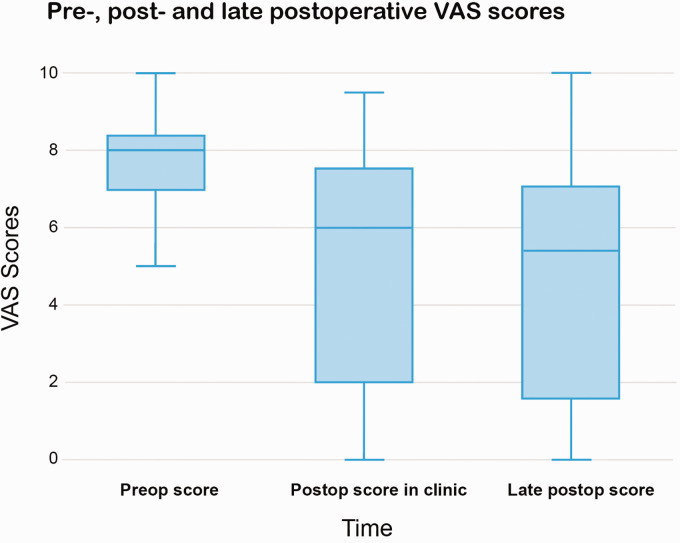
Preoperative, postoperative in clinic and late postoperative visual analogue scale scores.

The median preoperative VAS score for pain (*n* = 18) was 8.0 (range 5.0–10.0). The median postoperative VAS score at the clinic follow-up (*n* = 21) after a median follow-up of 8 months, was 6.0 (range 0.0–9.5). The median late postoperative VAS score obtained by the questionnaires (*n* = 18) was 5.4 (range 0.0–10.0), with a median follow-up of 3 years (range 1.0–7.0 years). The median VAS score decreased significantly postoperatively at follow-up in clinic (*p* = 0.013) and at the late postoperative follow-up (*p* = 0.035). The VAS score lowered in 14 patients and increased after surgery in 3/18 patients. Figure 2 shows the boxplots of preoperative, postoperative in clinic and late postoperative VAS scores.

There were no significant differences between patients with one or two surgeries before the reconstruction (*n* = 11) compared to patients with three or more previous surgeries (*n* = 11). The median postoperative clinic follow-up VAS scores were 6.3 for one or two surgeries and 2.0 for three or more (*p* = 0.387) and late postoperative scores were 6.4 and 1.7 (*p* = 0.153).

### Pre- and postoperative pain medication and treatment

Data on pre- and postoperative pain medication was available from 16 patients. The use of pain medication decreased postoperatively in 11 patients, remained the same in three and increased in one. One participant changed from opioid, amitriptyline and capsaicin cream to TENS and neurostimulation treatment. In total, 15 patients used some form of pain medication preoperatively, compared to nine patients after surgery. Six patients used a strong opioid such as morphine, oxycodone or fentanyl before surgery compared to none postoperatively.

### Patient-reported outcomes

The questionnaires were completed by 18 patients with a median time of 3.1 years (range 1.0–7.0) after surgery. No significant differences in SF-36 and EQ-5D-5L scores were seen in the unilateral compared to the bilateral neuropathic pain subgroups. Total MHOQ score as well as outcomes for overall hand function, daily activities, aesthetics, satisfaction and the total score were all significantly worse for the reconstruction side compared to the opposite side. SF-36, EQ-5D-5L, MHOQ and QuickDASH overall scores and MHOQ scores of the reconstruction side are presented in supplementary file Appendix S1.

## Discussion

Patients experiencing therapy-resistant ulnar nerve neuropathic pain after multiple failed surgical therapies pose a challenging problem. In these patients, extensive neurolysis with a subsequent free gracilis muscle flap coverage is a promising treatment option. Our results show a significant decrease in VAS scores at 8 months, followed by a moderate reduction and stability at long-term follow-up (median 3 years).

Chronic neuropathic pain has a severe impact on patients and diminishes health-related quality of life (Bailey et al., 2009; Girach et al., 2019). While not all patients experience pain relief or a reduction in pain, our cohort does show a decrease in VAS score with a mean pain reduction of 37% and 38% postoperatively in the clinic and at the late postoperative follow-up, respectively. A reduction of at least 32% is considered to be the minimum clinically important difference in chronic pain (Frahm Olsen et al., 2018). At the late postoperative follow-up, this was achieved in 7/15 patients. Only one study described the VAS score after neuropathic pain revision surgery using free gracilis muscle flap and supported our findings with a sustained reduction in pain at the 5.5-year follow-up (van Bekkum et al., 2020). A similar reduction in pain at short-term follow-up was found in a study on revision surgery for ulnar entrapment by subcutaneous transposition, submuscular transposition or neurolysis (Davidge et al., 2019). Stokvis et al. (2010a, 2010b) found location, duration of pain before treatment and the number of previous operations to be prognostic factors of surgical outcomes. Despite considerable variation, we did not find a significant correlation between median VAS scores and an increased number of surgeries before the gracilis reconstruction.

Reducing the usage of pain-relieving therapies and medications are an important aspect in patients experiencing chronic neuropathic pain. Therapy-resistant neuropathic pain is a well-known cause of opioid use, therefore contributing to the opioid crisis with all its consequences (Häuser et al., 2021; Helmerhorst et al., 2017). The use and misuse of these highly addictive drugs have an immense impact on national and global healthcare and the economy (Meyer et al., 2014); therefore, usage of opioids should be minimized. In our cohort, a decrease was seen in postoperative pain medication usage, specifically regarding opioid analgesics. All patients who required opioids (such as morphine, oxycodone or fentanyl) before surgery were completely free of opioid medications after surgery.

Our study reported significantly poorer scores on all domains of the SF-36 score when compared to the general Dutch population (Aaronson et al., 1998), confirming the negative impact on the quality of life of patients experiencing chronic neuropathic pain despite the postoperative improvement observed. The management of expectations is therefore a crucial part of the preoperative counselling required in order for patients to make a fully informed decision regarding revision surgery. Our patient-reported outcomes are comparable to outcomes reported in our earlier cohort (van Bekkum et al., 2020). As expected, patients with unilateral pain tend to score better on quality-of-life outcomes than patients with bilateral neuropathic pain of which only one side was surgically treated; however, the difference was not found to be significant, possibly due to the limited sample sizes (12 vs. 6 patients).

While outcomes regarding pain relief are promising, treatment of the chronic painful nerve with an extensive neurolysis and a subsequent free gracilis transfer is a complex surgical procedure with related risks and complications. In our cohort, no flap losses were seen and flap complications requiring surgery occurred in 2/22 operations. This is substantially lower compared to 4 and 8 out of 15 patients in the study by Van Bekkum et al., possibly as a result of a learning curve (van Bekkum et al., 2020). Our complication rates are comparable to other studies on free gracilis muscle reconstructions (Franco et al., 2017; Osiogo et al., 2006; Redett et al., 2000).

Nerve coverage with free flaps is considered to be the last possible surgical treatment option aimed at pain relief (Holmberg and Ekerot, 1993). Although the exact pathophysiology of a free flap in case of neuropathic pain is still unknown, mechanisms described to contribute to the beneficial effects include the following (1) a new blood supply, possibly promoting revascularization of a devascularized segment of the nerve; (2) excision of the hyperaesthetic area of skin; (3) tissue padding, which may insulate the nerve from the traction forces of adjacent moving tendons and function as a cushion from external pressure on the overlying skin; and (4) creation of an environment with less scar tissue. Though all surgeries heal with scar tissue, the rationale is that the interface between the well-vascularized flap and the nerve may result in less fibrosis and therefore allow improved longitudinal gliding of the nerve (Holmberg and Ekerot, 1993; Millesi et al., 1990).

Our study was limited by the retrospective design and therefore unavoidable missing data of pre- and postoperative VAS scores. A further limitation is the lack of preoperative PROMs with which to compare outcome data. However, our postoperative PROMs are presented to allow for comparison with future studies.

## Conclusion

In the treatment for therapy-resistant neuropathic pain of the ulnar nerve, extensive neurolysis and subsequent free gracilis muscle flap coverage show promising results on pain reduction. As the main objective of the procedure, a decrease in VAS scores is seen after surgery, which remains lowered during long-term follow-up. Postoperatively, a reduction in the use of pain medication is observed in 11/16 patients of the cohort and all patients who required preoperative opioid analgesics were opioid-free after surgery.
